# Supplementary material for: Necroptosis Mediates Muscle Protein Degradation in a Cachexia Model of Weanling Pig with Lipopolysaccharide Challenge
Source: Int J Mol Sci. 2023 Jun 30;24(13):10923. doi: 10.3390/ijms241310923 (PMC10341553; doi:10.3390/ijms241310923)
Supplement: Supplementary file 1 [file ijms-24-10923-s001.zip › ijms-2408771-supplementary.pdf]

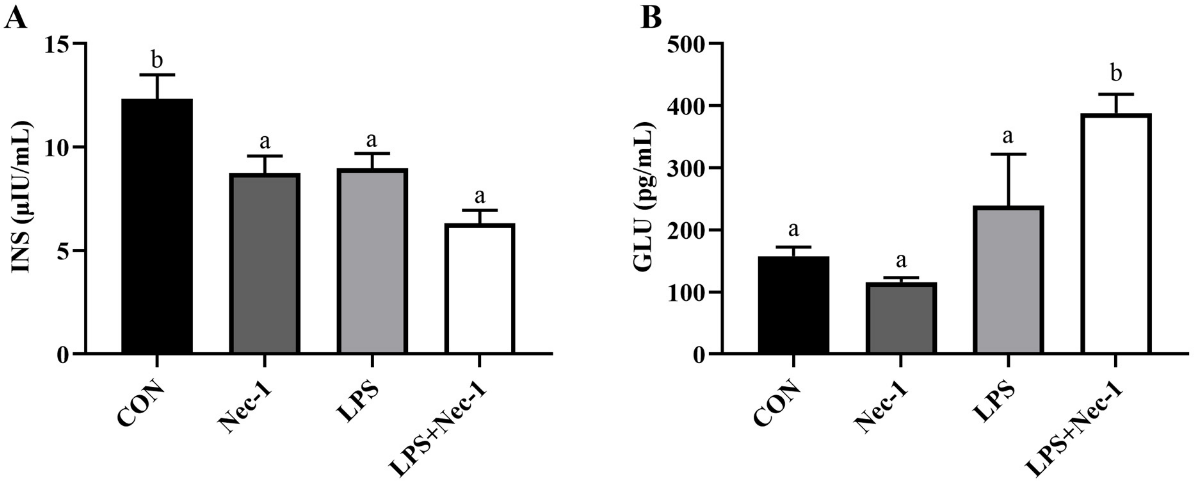

Supplementary Figure S1. Effects of inhibition of necroptosis by Nec-1 on INS and GLU concentrations in the plasma. The piglets were pretreated with Nec-1 or DMSO for 30 min and then injected with saline or LPS for 4 h. (**A**,**B**) The concentrations of INS and GLU in the plasma. Data are presented as the means ± SEM, *n* = 6. [ab] Means without a common letter differ ($p < 0.05$).
